# UK government’s new placement legislation is a ‘good first step’: a rapid qualitative analysis of consumer, business, enforcement and health stakeholder perspectives

**DOI:** 10.1186/s12916-023-02726-9

**Published:** 2023-01-26

**Authors:** Sarah Muir, Preeti Dhuria, Emma Roe, Wendy Lawrence, Janis Baird, Christina Vogel

**Affiliations:** 1 Medical Research Council Lifecourse Epidemiology Centre, University of Southampton, Southampton General Hospital, Tremona Road, Southampton, SO16 6YD UK; 2 School of Geography and Environmental Science, University of Southampton, Highfield Campus, Southampton, SO17 1BJ UK; 3 National Institute for Health Research Southampton Biomedical Research Centre, University of Southampton and University Hospital Southampton NHS Foundation Trust, Tremona Road, Southampton, SO16 6YD UK; 4 NIHR Applied Research Collaboration Wessex, Southampton Science Park, Innovation Centre, 2 Venture Road, Chilworth, Southampton, SO16 7NP UK; 5 Centre for Food Policy, City, University of London, Northampton Square, London, EC1V 0HB UK

**Keywords:** Food policy, Placement legislation, Qualitative research, Stakeholder views, Retail food environment, HFSS foods

## Abstract

**Background:**

The current food system in England promotes a population diet that is high in fat, sugar and salt (HFSS). To address this, the UK government has implemented legislation to restrict the promotion of HFSS products in prominent locations (e.g. store entrances, checkouts) in qualifying retailers since October 2022. This study investigated the perceived impact of the legislation for affected stakeholders.

**Methods:**

A pre-implementation rapid qualitative evaluation of stakeholder interviews. One hundred eight UK stakeholders participated in the study including 34 consumers, 24 manufacturers and retailers, 22 local authority enforcement officers and 28 academic and charitable health representatives. A participatory conference was used to enable policy recommendations to be confirmed by stakeholders.

**Results:**

Stakeholders perceived the legislation to be a ‘good first step’ towards improving population diet but recognised this needed to be considered amongst a range of long-term obesity policies. Areas of further support were identified and these are presented as six recommendations for government to support the successful implementation of the legislation: (1) provide a free central HFSS calculator, (2) refine legislation to enhance intent and clarity, (3) conduct a robust evaluation to assess intended and unintended outcomes, (4) provide greater support for smaller businesses, (5) provide ring-fenced resources to local authorities and (6) create and communicate a long-term roadmap for food and health.

**Conclusions:**

This legislation has the potential to reduce impulse HFSS purchases and makes a solid start towards creating healthier retail outlets for consumers. Immediate government actions to create a freely accessible HFSS calculator, support smaller businesses and provide additional resources to local authorities would support successful implementation and enforcement. Independent evaluation of the implementation of the legislation will enable monitoring of potential unintended consequences identified in this study and support refinement of the legislation. A long-term roadmap is necessary to outline strategies to support equal access to healthier and sustainable food across the whole food system within the next 20–30 years.

**Supplementary Information:**

The online version contains supplementary material available at 10.1186/s12916-023-02726-9.

## Background

The UK food system is currently set up to promote an unhealthy and unsustainable population diet that increasingly cultivates unaffordable healthy options for consumers, rising inequalities in obesity and detrimental planetary impact [1, 2]. This situation was eloquently described and labelled the ‘junk food cycle’ in the UK’s independent National Food Strategy [1], a government-commissioned review led by Henry Dimbleby which investigated how the food system works, the damage it is doing to human and environmental health, and key interventions needed to prevent these harms. The review’s final report described several ‘destructive feedback loops’ within the food system which are driven by a highly competitive market and cheap unhealthy ingredients that encourage food companies to invest in and develop high numbers of high fat, sugar and salt (HFSS) and ultra-processed foods. A majority of consumers show a preference for these nutritionally poor, energy-dense products because they are affordable, available, convenient and widely marketed, which in turn perpetuates further investment, production and overconsumption. To provide all households in the UK an equal chance at achieving and sustaining good health, the National Food Strategy stressed the need for policies which focus on changing the source of the problem, specifically food environments and the food system.

Retailers design supermarkets to maximise sales and profits by using prominent positioning (e.g. displays at store entrances, aisle ends and checkouts) or price promotions which can weaken shoppers’ good intentions to choose healthier foods [3]. Products high in HFSS are more often promoted in prominent high-traffic locations within grocery settings than healthier products [4–6]. Scientific evidence shows that increased exposure to prominent displays and price promotions on HFSS is associated with poorer dietary-related choices [7, 8], as well as increased BMI [9] and health inequalities [10, 11]. Intervention research, however, shows that creating healthier layouts in retail businesses can improve consumer food purchasing and consumption patterns [12–14]. For example, placing fewer unhealthy foods at checkouts led to a 15.5% reduction in purchases of these items and a 76% self-reported reduction in snacking on items bought at checkouts [15]. Similarly, placing fruit and vegetables at store entrances and removing confectionary and other HFSS products from checkouts increased and reduced sales and consumption of these product categories, respectively [12]. The impact of these placement changes on population health is likely to be more potent and equitable amongst businesses if they were legislated [16].

The UK government is the first country to legislate to improve retailers’ food marketing strategies. Since 1 October 2022, retail businesses can no longer place HFSS items in prominent locations (e.g. store entrances, end-of-aisles, checkouts, designated queuing areas or online homepages or checkout pages) [17]. Qualifying businesses include those with over 50 employers and premises that are >2000 square feet including supermarkets, franchise convenience stores and non-food retailers that sell items such as confectionary at checkouts. Small independent corner stores, specialist retailers (e.g. a chocolatier) and those in the out-of-home sector (e.g. cafes, cinemas) do not need to comply. Food and drink are in-scope of the legislation if they are prepacked (i.e. not loose bakery or confectionery items), fit into one of thirteen product categories (including soft drinks, savoury snacks, breakfast cereals, confectionary, ice cream and lollies, cakes and cupcakes, sweet biscuits and bars, morning goods, desserts and puddings, sweetened yoghurt, pizza, potato products and prepared meals, products in sauce and breaded or battered foods) and score four or more on the UK nutrient profiling model. Trading standards officers or environmental health officers within local authorities are responsible for enforcing the regulations. The legislation also bans the use of volume-based promotions (i.e. multibuy promotions) on HFSS products but, due to concerns about the cost of living crisis, implementation of this component has been delayed until October 2023 [18, 19].

In order to understand how public health policies are implemented and their impacts in real-world environments, they need to be considered in the context of the broader system in which they take place [20]. Adopting a complex systems perspective to policy evaluation provides a focus on the interplay between the policy and its surrounding system which helps identify emerging consequences that may amplify or dampen intended impacts and inform future decisions about policy implementation. Qualitative research methods are particularly useful for unpacking complexity because they enable exploration of the various elements, structures and relationships within the system that is affected by a policy [21].

A pre-implementation complex systems evaluation of stakeholders’ perspectives on this novel legislation is necessary to provide policymakers with clear recommendations on additional actions needed for effective implementation and enforcement. Consistent implementation and enforcement will help ensure the legislation achieves its intended impact of improving the population diet and reducing obesity levels, particularly amongst children [22]. To the authors’ knowledge, no study to date has conducted a comprehensive pre-implementation evaluation which incorporates views from a range of stakeholders from across the system affected by this legislation. To address this evidence gap, this study applied rapid qualitative methods to allow for time-sensitive data collection from a large number of participants [23, 24] and participatory techniques to confirm rapid findings [25]. More specifically, this evaluation applied complex systems methods to explore stakeholder’s underlying assumptions of the legislation, views on support required for effective implementation and enforcement and opinions on policy and evaluation recommendations.

## Methods

### Study aim, design and setting

The study aimed to (i) assess stakeholders’ views on the legislation, including their perceived benefits, concerns and support needs arising from its implementation, using a pre-implementation rapid qualitative evaluation, and (ii) determine and prioritise recommendations for policy, using participatory techniques. The setting for this study was England, UK.

### Participants

A purposive sample of four stakeholder groups affected by the legislation was recruited including consumers, businesses (including retailers, manufacturers and wholesalers), enforcers (including a mix of trading standards and environmental health officers from unitary and two-tiered local authorities from northern, central and southern regions of England) and health representatives (i.e. academics working in the public health field at a number of UK universities and non-government charitable organisations focused on improving population diet or reducing diet-related noncommunicable diseases with headquarters in England). Recruitment followed two phases.

In phase 1, women consumers who held and used loyalty cards at stores taking part in the ‘Women’s Responses to Adjusted Product Placement and its Effects on Diet’ (WRAPPED) study and who had previously accepted the personalised postal letter to participate in the WRAPPED study were sent an additional invitation letter via post to participate in a semi-structured interview for an additional £10 incentive. Interested participants contacted the research team to schedule a telephone interview. Further details about the WRAPPED study can be found elsewhere [26]. The sample of WRAPPED participants approached included 40 women purposively selected to cover approximately equal representation from intervention and control stores, northern and southern regions of England, low and higher education levels and households with and without young children. Women were the focus of this phase because they represent an important target group for improving the diets of the broader population. Women continue to dominate household decisions about food shopping and preparation which influences the short- and long-term health of their family members [27, 28].

In phase 2, business, enforcement and health participants were recruited via email or in-person using various strategies, including existing professional contacts, attendance at retail and trading standards meetings, desk-top identification using publicly available websites or government consultation responses, trading standards email lists and newsletters and snowball sampling where participants introduced the research team to potential participants.

### Data collection

Data were collected in two phases.

#### Phase 1: Consumer interviews

Consumer interviews were conducted by PD (registered public health nutritionist) via telephone in May 2020 between the two public consultations about the regulation and prior to its enactment. Interviews were already ongoing as part of WRAPPED and provided an opportunity to also ask about the planned legislation.

#### Phase 2: Business, enforcer and health stakeholder interviews

Semi-structured interviews with business, enforcer and health stakeholders were conducted by SM (experienced qualitative researcher with a psychology background) and PD. Data were collected between August 2021 and April 2022, following the release of the draft legislation (July 2021) and prior to the publication of detailed policy guidance (April 2022). Individual interviews were held with business and enforcement stakeholders, but in some cases, two members of the same business or the same local authority took part in a joint interview. Health representatives took part in focus groups containing a mix of academic and health charity representatives or individual/paired interviews. Focus groups were conducted by PD and observed by SM. All interviews were conducted using video conferencing software (Microsoft Teams/Zoom).

#### Interview guides

Semi-structured interview guides were developed for each stakeholder group (see Additional file 1). This approach enables topics to be explored systematically whilst allowing participants the freedom to discuss issues relevant to them [29]. Consumers were asked questions about how their shopping habits in supermarkets could change if the government enacted the legislation under consideration. Phase 2 participants were asked questions about their views of the legislation, including perceived benefits, concerns and support needed for successful implementation. Health stakeholders were also asked for their views on how the legislation may impact health inequalities (a priority area for the UK government) and methods for evaluating the legislation’s effectiveness on diet and health.

### Data analysis

#### Phase 1 analysis

Transcriptions of audio files were created by a professional transcription company. Data were analysed by SM using the six-step process of thematic analysis detailed by Braun and Clarke. Themes were reviewed, defined and named with team members (CV, PD) in a series of team meetings in accordance with steps four and five of thematic analysis. PD checked the coding of 10% of transcripts to validate themes—there were no discrepancies between both team members’ analyses.

In June 2022, final themes from the consumer stakeholder group and interpretations of these themes were confirmed in a participatory discussion [25] with the WRAPPED study patient and public involvement (PPI) panel.

#### Phase 2 analysis

Rapid qualitative evaluation was used in phase 2 to enable the research team to use a team approach to collect valid, timely results from a large number of stakeholders. Rapid approaches are particularly suited for time-sensitive policy studies to allow results to be shared with policymakers as they arise [23, 24] and results have been found to be comparable to more established qualitative approaches [30, 31]. Interviews were video recorded (but not transcribed), and summaries of all main points were made by the researcher who conducted the interview/observed the focus group (SM or PD) and after listening back to the video file [32]. Data from each summary were entered into an overarching Rapid Assessment sheet (RAP sheet) detailing the summary points from all participants [23, 30]. Initially, a separate RAP sheet was created for each stakeholder group. Each stakeholder RAP sheet was sectioned into categories labelled (i) ‘benefits’, (ii) ‘concerns’ and (iii) ‘support needs’. The final section was labelled ‘implementation’ for businesses, ‘enforcement’ for enforcers and ‘evaluation’ for retailers. Under the benefits, concerns and support needs, there were subheadings to enable issues related to consumers, businesses and enforcement to be detailed. This activity highlighted the presence of common latent themes across stakeholder groups. Consequently, latent theme labels were applied across the RAP sheets that incorporated each stakeholders’ views. Regular meetings were held between SM, PD and CV during the data collection period to discuss and refine themes, considering the system context within which the legislation is being implemented. Brief reports of emerging findings were disseminated to UK government policymakers. Many themes found in phase 2 linked closely with those raised by consumers in phase 1; thus, to provide an overview of all stakeholders affected by legislation, views from both phases are presented collectively in the ‘Results’ section.

Policy recommendations were generated by (i) explicit requests for support needs from participants and (ii) interpretation of resulting themes and sub-themes through research team discussions which adopted a complex systems perspective. In May 2022, the research team, in partnership with the Consumer Goods Forum and Chartered Trading Standards Institute, held a virtual half-day conference on the topic of supporting the successful implementation and enforcement of the legislation [33]. The conference was promoted through the research teams’ and our partners’ professional networks and via Linkedin and Twitter (enabling speakers and other interested parties to share the registration link). Delegates registered for the conference on Eventbrite. Over 450 business, enforcement and policy stakeholders registered and 345 attended on the day. The conference included six presentations aimed to improve understanding of the regulation’s purpose and scope. Representatives from each conference partner, the Food Foundation, the British Retail Consortium and the Association of Convenience Stores delivered presentations. The conference was also used to conduct participatory research activities with stakeholders [25] by sharing our research team’s preliminary results and asking delegates to actively confirm and rank the six key policy recommendations resulting from the research in order of perceived importance. The poll function on Zoom allowed participants to provide their rank anonymously and see the overall results live on screen.

## Results

### Participants

A total of 108 stakeholders were interviewed (see Additional file 2). The consumer group included 34 women who primarily shopped at discount supermarkets with less healthy in-store environments [5], and mostly had lower levels of educational attainment (Additional file 2: Table S1). The business group consisted of 24 participants including 15 retailers from the supermarket, convenience store, online and non-food retailers and their trade representatives and 9 manufacturers or wholesalers who supplied these retailers, along with their trade representatives (Additional file 2: Table S2). A total of 22 enforcement stakeholders (including some primary authorities who advise large retail businesses on legislation) across a range of northern and southern regions in the UK participated. This sample consisted of 13 trading standards officers and their organisational representatives, 6 environmental officers and their associated professional organisations and 3 public health officers (Additional file 2: Table S3). Enforcement is more likely to be carried out by trading standards officers but as decisions about enforcement are made at a local level some authorities may also involve environmental health officers. Overall, 28 health representatives participated in the study consisting of 9 world-renowned public health/food policy academics and 19 representatives from health charities involved in lobbying about food policy and/or campaigning for action on obesity-related illness (Additional file 2: Table S4).

### Aim 1: Rapid analysis of stakeholders’ views on the legislation

The rapid analysis identified two key themes. Theme 1, ‘This legislation is a “good first step” but there is potential for unintended consequences’, incorporates perspectives from the four stakeholder groups. Theme 2, ‘Inconsistent approaches may affect legislation impact’, highlights the concerns and support needs of business, enforcement and, to a lesser extent, health stakeholders. These two themes are detailed below with verbatim quotes from participants to illustrate. The six policy recommendations that were informed by these results and validated with conference delegates are subsequently presented.

#### Theme 1: This legislation is a ‘good first step’ but there is potential for unintended consequences

##### 1a. Real hope for positive impact on health

Stakeholders from all groups believed that the legislation has great potential to (i) reduce impulse purchases of HFSS products, (ii) increase opportunities to promote healthier foods in prominent locations and (iii) drive cultural change about food shopping environments that could increase consumer demand for healthier foods. There were, however, concerns that the continued availability of HFSS products in retail stores will weaken the impact of this legislation and result in consumers simply changing their journeys through stores without altering purchasing patterns.

I do think it [legislation] would be good for the general health of the public if the temptation wasn’t there as you walked in shops and at the checkouts as well because when you’re queuing the children tend to fiddle. It would help families more if them products were a bit more out of sight. (6054, Consumer).

If it works then the placement of these rich delicious goods won't be in the footfall places where people just throw a cheeky Twix into the trolley to have on the way to the car. I think it will influence behaviour like that by taking away the choice and the opportunity. [ … ] or people will just learn and will just pick up the Twix earlier on in the shopping. (13012, Enforcer, Environmental health).

##### 1b. Impact of exemptions on health and inequalities

Product exemptions (e.g. products not prepacked for direct sale like doughnuts/pick-and-mix sweets and alcohol), promotion exemptions (e.g. meal deals, reduced price promotions) and omissions of prominent promotional spaces (e.g. middle-aisle baskets, gondolas) have resulted in genuine concern from retailers, enforcers and health stakeholders that retailers will exploit these loopholes and subsequently undermine the legislation’s health aims.

Something we’ve called for is no exemption for unpacked foods because it’s the same number of calories regardless of the packaging that they’re in. Alcohol is totally exempt, pies are totally exempt, and these do actually make quite a strong contribution to total calorie intakes. (11012, Business, Retailer).

Will the problem shift so we will potentially see a huge amount of 25% off or 50% off price promotions instead? (13013, Enforcer, Trading standards).

Across stakeholder groups, there was concern that the exemption of businesses with less than 50 employees and stores smaller than 2000 square feet has real potential to increase health inequalities. Enforcer and health stakeholders highlighted that consumers who rely on these smaller stores often have lower incomes, or are younger or older adults. These groups are known to have less healthy diets and poorer access to larger supermarkets. Some study participants expressed the importance of including all business types (both small and large) in the legislation.

The sorts of people that are going to benefit from the impact are the people that shop in larger supermarkets, which are more likely to be our more affluent residents. From a health inequalities angle, that’s why it will be important to start trying to shake the smaller businesses down the line. (13037, Enforcer, Public Health).

There shouldn’t be any exceptions, everyone should have to follow it whatever size of the business so that it becomes a social norm then so that it actually feels quite weird if anyone isn’t following the rules. (12003, Health, Academic).

##### 1c. Healthy products remain less affordable than unhealthy products

There were mixed views about the impact the legislation could have on food shopping affordability. Retailer, enforcer and some consumers (particularly those who described feeling tempted by impulse purchases and were less likely to use a shopping list) expressed concern that the legislation could increase household food shopping costs if multibuy promotions were no longer available. Some also expressed concern about the negative effect on business profits which could further drive up food costs for consumers.

Whenever new regulations come in, then there’s obviously some pass through of that cost to consumers, and we’re seeing the pass through of costs because of cost pressures in the business, which are really significant. (11037, Business, Retailer).

I think prices will go up [if multibuys were banned], if you need 12 chocolate bars ‘cause you’ve got a large family, you’re paying more for it eventually, aren’t you? (13012, Enforcer, Environmental health).

Conversely, health representatives believed removing value-based promotions on HFSS products could improve food costs for consumers. Similarly, some consumers who planned their shopping and felt less tempted by promotions believed there would be little effect on their shopping habits because HFSS was only bought as a treat.

Far from saving people money promotions lead to more purchases and contribute to greater consumption, resulting in overweight and obesity. That’s the bottom line. (12039, Health, Charity).

There are certain things that I do buy as treats for my children but like I said they’re treats so if they’re not on offer then I would still get them but probably not as frequent. (6029, Consumer).

Nonetheless, many consumers, health representatives and some retailers raised concerns about the affordability gap between HFSS and healthier products. They felt it was an essential issue for policymakers to address in future policies because reducing price promotions on HFSS will not automatically make healthier products more affordable.

I would advise the government they need to provide the supermarket with the lower prices for fresh fruit and veg, so we can then afford it as well. No point in them discouraging the non-healthy food when the healthy food prices are going really high. (6301, Consumer).

Sharon Hodgson (Member of Parliament) actually phrased this really well today and to use her words: ‘It’s a stark reality that the cheapest food is often the most calorific. It’s far more expensive to fill up hungry children with healthy food. To give an example for chocolate muffins for a pound in a supermarket, 6 apples are usually £2.00.’ It’s that price discrepancy that we know drives a lot of purchasing and leads to the fact if you’re living in a deprived area, then you’re twice as likely to have obesity. I’d be really worried that the price gap is not levelled and ideally that would be something that is monitored as part of the evaluation. (12055, Health, Charity).

##### 1d. Legislation is only one part of a bigger strategy

Each stakeholder group felt this HFSS regulation formed an important first step of a broader policy agenda to address obesity and poor diet, but alone it would be insufficient to solve these issues. Stakeholders felt a clear, comprehensive, long-term policy strategy is needed.

This [is] a step in the right direction but it is one of many policies. We talk about a comprehensive strategy and sort of coherent policy landscape to make sure that the availability and visibility of HFSS foods is limited (part of) a larger shift towards greater accessibility, affordability and visibility of healthier options so that health is the default, and it isn’t about individuals having to make that choice, it’s about the environment that provides that. (12035, Health, Charity).

Hopefully it achieves its goal, not necessarily on its own, but as one element within a package of public health measures to help customers make the healthier choice whether it’s on its own or as a mix of elements. (11004, Business, Retailer).

Manufacturers expressed that the lack of clear, long-term policy direction on food and health from the government made it difficult for them to set their strategic business priorities. Some have spent time and resources following recommendations to reduce portion size or reformulate products to reduce sugar or salt. These efforts, however, do not align with the requirements of the nutrient profile model which forms a key part of this legislation.

I’d be interested to know what their [the Government’s] long-term plan is. It doesn’t affect all categories now, I don’t know whether they would ever roll it out to more categories in the future. Also we know there’s HFSS2, that was a new nutrient profile that was out for consultation years ago. And as far as I know Government have signed it off internally but they’ve never come out...a new profile would be more restrictive again. I guess it would be disappointing to see that come out when a lot of work has been done to reformulate for this profile. (11079, Business, Manufacturer).

#### Theme 2: Inconsistent approaches may affect legislation impact

##### 2a. Legislation complexity and ambiguity leave room for interpretation

Stakeholders felt this legislation was complex and ambiguous, making it difficult to implement and open to interpretation. There were concerns that the complexity increased the likelihood of inconsistent implementation and enforcement across store types and geographical regions. As participant 11011 describes below, stakeholders felt this ambiguity in the written regulation could have been avoided if stakeholders’ concerns had been considered more thoroughly. Furthermore, enforcement officers, including 13023, explained that inspection visits to stores may require individual interpretation (i.e. a ‘designated queuing area’, ‘aisle-end’ or ‘meal deal’ may be interpreted differently by retailers and may not match definitions in the regulations).

There is a lack of clarity and forethought on writing the regulations, and a consistent refusal to listen to industry and understand the challenges. (11011, Business, Retailer).

A lot of situations are unclear, and they don’t fall neatly within what is written and then it’s up to enforcers and businesses to look at where their situation falls in and interpret the guidance of the legislation accordingly. (13023, Enforcer, Trading standards).

The nutrient profile model used to define in-scope products is complicated and results in products within included product categories being unexpectedly exempt (e.g. high-protein pizzas, high-fruit buns) which could make enforcement difficult. Retailers were concerned that product definition complexities and their dependence on accurate scores from manufacturers will make them liable for non-compliance.

All the retailers are reliant on colleagues in a store making sure they are fully adhering to the rules which in some areas don’t make a huge amount of sense to the general person scoring goods. For example, a meat lovers’ pizza is non-HFSS on the basis that it has a high quantity of protein. (11004, Business, Retail).

There’s such variety, and [you need to consider, for example] how many nuts are in it and all the rest of it, when we get down to those kind of discussions, that creates huge problems for regulators, because you don’t want to be arguing over whether a product’s in scope or out of scope, you want it to be very clear. (13011, Enforcer, Trading standards).

##### 2b. Differences in prioritisation of the legislation

Inconsistencies in the level of prioritisation retailers and enforcers will give this legislation were clear. Some businesses were already making changes, hoping to gain a competitive edge on promoting healthy choices and accelerating existing health-related business plans. Other businesses, however, were focused on making changes to ensure compliance, whilst exploring ways to ensure profits from HFSS products were not affected.

We feel like this is an opportunity to differentiate ourselves if we can do it well, you know, make it better for people. [ … ] there could be that rush to the top as it were, instead of the bottom where we’re all trying to find new ways to promote healthy stuff. (11011, Business, Retailer).

We will still sell the same range of confectionery it just won’t be on an end facing a till. But I will make sure that it’s as close to a till that is legally compliant. (10000, Business, Retailer).

Regional differences in enforcement approaches are also likely according to local authorities’ prioritisation of healthy eating and obesity as a public health concern. This could result in somewhat patchy enforcement activities.

You could have a situation of Councillor X who becomes leader says, “I really believe in child health, and I want to promote this,” and will drive it. But in other authorities, they might say, “No, we’ve got no time for that, we’ve got all this to deal with.” (13003, Enforcer, Trading standards).

##### 2c. Differences in resource availability and capacity

Understanding the legislation, determining which businesses qualify and making changes to shop infrastructure has been time-intense and costly for retailers. Larger businesses have had better access to legal support (i.e. through primary authority links) and will be less affected by short-term profit loss and implementation costs than smaller stores. The quote below from an independent retailer illustrates this point clearly:

We do promotions because of attracting customers in the shop, to come to buy stuff. If we can’t do it, they have that mentality to go to a supermarket. Obviously we’re losing customers. Prices going up, wages going up, electricity bills going up. Everything’s going up, if we’re not getting customers the way we used to be getting, how are we going to survive? (11086, Business, Retailer).

Generally you can be pretty sure that an independent’s due diligence procedures won’t be anywhere near as robust as a national supermarket, particularly regarding training [ … ] They’re focused very much on the business, [the] sale of products and less so on diligence. And that reflects in the kind of problems that we [environmental health] get. (13015, Enforcer, Environmental Health).

Enforcement stakeholders clearly described how resource issues will mean the majority of local authorities cannot prioritise this legislation. Limited staff and funding cuts result in prioritisation of immediate risks to health (e.g. allergens, crime, safety).

And when you’re making the decision between do we try and deal with this unsafe chainsaw that could kill somebody today, or do we deal with these products that contribute to obesity, that is a long term issue, it’s very difficult to get priority for long term issues over immediate short term issues. You’ve got limited resources, you have to target those at stuff that has probably the most imminent risk. And this (legislation) doesn’t fall into that category. (13011, Enforcer, Trading standards).

### Aim 2: Determination and prioritisation of policy recommendations

**Fig. 1:**
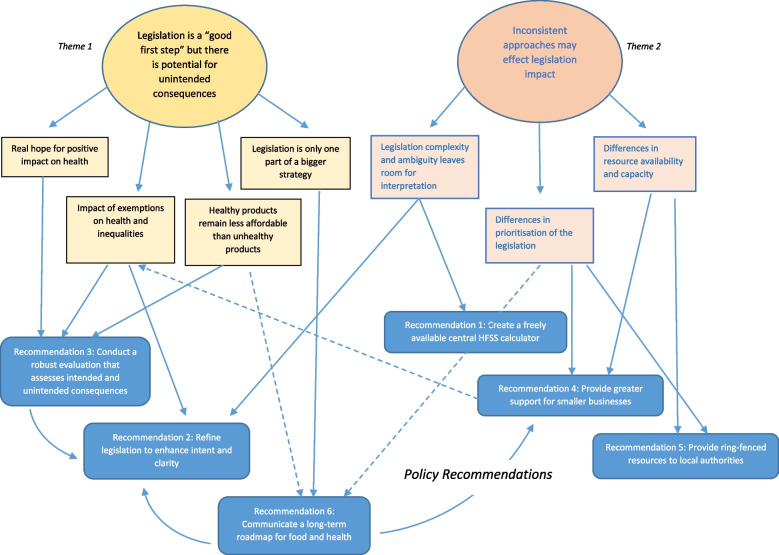
Links between stakeholder sub-themes and the development of six policy recommendations to optimise legislation’s intended impacts

Figure 1 illustrates how the identified themes and sub-themes informed the development of the six recommendations for policymakers. If acted upon, these recommendations could help to overcome issues of inconsistent legislation implementation and minimise unintended consequences, particularly inequalities in health and commercial competition. The numbering of these recommendations represents the priority order for stakeholders according to the results of the priority setting activity undertaken by conference delegates. Detailed descriptions of these recommendations, alongside stakeholder quotes supporting their development, are shown in Table 1.

**Table 1:** Six policy recommendations based on stakeholder views

Policy recommendation	Illustrative quote
**1. Make a central HFSS calculator freely available**	
All business staff (managers, employees), business types (retailers, manufacturers, wholesalers) and enforcers require access to a free, accurate and mobile HFSS calculator. It should be inclusive of cultural foods and regularly updated to ensure consistent, effective implementation and enforcement of this legislation.	‘Everyone wants the government to have a way of centrally holding the nutrient profiling scores for products (i.e., composition of foods database) so that retailers and enforcers can access it. Government can do more to facilitate information about what is an unhealthy food.’ (11007, Business, Retailer)
**2. Refine legislation to enhance intent and clarity**	
Details about the purpose of the legislation could improve understanding and receptivity to comply within the spirit of the legislation. In particular, the regulations should add details about which products *can* or *should* be promoted, how businesses can determine products’ HFSS scores and how enforcers obtain information to define a qualifying business. Using evidence from thorough legislation evaluation (recommendation 3) is critical to inform legislation refinement to improve its effectiveness.	‘The way the UK regulations are written a lot of the time are in a very literal style that sets out a number of rules but if you look at many of the EU regulations, they have this introductory text that sets out what the point is and what the purpose of the regulations are, and under EU regulations there is a requirement for you to interpret the legislation with regard to its intention. The [UK] approach can be very literal in terms of what does the regulations say, and if it doesn’t say that, then you’re allowed to do it [ … ] but maybe some introductory texts to cut out any loopholes.’ (13013, Enforcer, Trading Standards)
**3. Conduct a robust evaluation to assess intended and unintended consequences**	
Short-term evaluation should assess (i) legislation implementation and enforcement activities across all business types; (ii) changes in sales and purchasing patterns across all HFSS categories and alternative products (fruit, vegetable, alcohol etc.) in qualifying and exempt businesses; and (iii) differences by consumer and regional demographics to examine the impact on inequalities. Long-term evaluation should assess changes in societal attitudes, dietary patterns and obesity rates, as well as business outcomes and broader food system changes.	‘The main thing for us is monitoring data that’s collected through health services, particularly around children, young people, and regular weighing and things like that. You can look at the amount of these products that are sold as well to monitor whether or not that goes up or down, whether or not there appear to be shifts. Supermarkets have amazing data that can drill right down to individuals. Obviously the weight thing is a bit of a longer term, but you could immediately monitor the sale of these products particularly in relation to healthier products.’ (12042, Health, Charity)
**4. Provide greater support for smaller businesses**	
Specific guidance for smaller businesses that is culturally accessible and additional funding for local authorities to support smaller retailers who do not have in-house legal support would increase awareness and compliance amongst all store types. Incentives for small retailers and their suppliers to improve the healthfulness of the foods they offer could be considered, in recognition of their small profit margins and low customer demand for healthy foods.	‘Smaller businesses do need more support in understanding the legislation [and] some funding for supporting those smaller businesses. And some very clear guidance, aimed at smaller shops because they’ve got a smaller floor area, they’ve got narrow aisles, they’ve got end of aisles nearer to tills. [Previously] the Government funded for business support visits. That would help enormously. But I’m not going to hold my breath for that one … ’ (13039, Enforcer, Environmental Health)
**5. Provide ring-fenced resources to local authorities**	
Additional resources for local authorities are essential to enable enforcers to become familiar with the new legislation, to provide support to all business types and to make visits to premises. This resource could be provided in the form of government funding for local authorities to conduct a HFSS legislation-specific project with targeted outcomes.	‘We’ve not got the resource to go and do the things on a daily basis that we desperately need to do. I suppose eventually, if we’ve got the scope to do it we’ll possibly pick it up as a project, a bit of project work to see what compliance rates are like.’ (13034, Enforcer, Trading standards)
**6. Create and communicate a long-term roadmap for food and health**	
A long-term roadmap for food and health is necessary to unify stakeholders’ priorities and timescales around the required action to achieve the collective desire for a food system that ensures healthy, sustainable diets for all within the next 20–30 years. It should build on work invested into Henry Dimbleby’s National Food Strategy review and the Obesity Health Alliance’s 10-year healthy weight strategy and be led by a statutory body such as the Food Standards Agency.	‘It’s just trying to find the sweet spot of, you know, you’ve had the Public Health England targets for a reduction in salt, for example. You’ve then got the HFSS now, you’ve got traffic light labelling that we look at, and then we’ve got our internal reformulation criteria as well. So it’s working within all of those, which can be quite complex. [ … ] [an] other thing we do look at is portion size.[ … ] It doesn’t change the score because it’s based on 100 grams but it’s one of those other things that we do because it's the right thing to do to offer consumer choice.’ (11079, Business, Manufacturer)

Three recommendations are for immediate action to enable effective implementation and enforcement of the legislation, namely (i) provision of a free central HFSS calculator to support consistent and accurate identification of in-scope products across store types and regions, (ii) providing additional ring-fenced resources to local authorities to ensure support is offered to all business types and enforcement activities are conducted consistently across geographical regions and (iii) providing greater guidance and support to smaller businesses to enable them to comply.

The remaining three recommendations are medium- to long-term actions that aim to help optimise the intended impact of the legislation, including (i) conducting a robust evaluation to assess implementation and enforcement, as well as outcomes of food purchasing, diet and obesity patterns across consumer groups, business types and regions; (ii) applying evaluation data to refine the legislation to facilitate consistent implementation and enforcement within the spirt of improving public health; and (iii) the creation of a long-term roadmap for food and health that unifies stakeholders on a strategy and timeframe to achieve a food system supportive of a healthy, sustainable population diet for all.

## Discussion

### Summary of findings

This study is extremely timely and demonstrates unique and valuable insights regarding the perceived intended benefits of this novel legislation, as well as highlighting potential unintended consequences resulting from inconsistencies in implementation. All stakeholders welcomed the legislation as a ‘good first step’, but with their expertise and understanding of the food retail market and enforcement practices were able to share valuable feedback about the complexity of the current legislation, propensity of loopholes in the guidance and why they consider that the exemption of some businesses could compromise health and business outcomes. Stakeholders anticipate that implementation and enforcement of this HFSS legislation are likely to be patchy. This situation is a consequence of considerable variation in engagement between store types and levels of prioritisation and resource availability to the legislation differ across regions. Targeted support from the government for smaller businesses and local authorities, alongside endorsement of a freely available HFSS calculator, could help to optimise implementation and public health benefits. Furthermore, across stakeholder groups, there was widespread recognition that this legislation would best achieve its intended aims of shifting the whole population dietary patterns to reduce obesity if it was (i) thoroughly evaluated, (ii) refined over time and (iii) implemented alongside a coherent range of other complementary policies.

### Comparison with previous research

This study showed that, across stakeholder groups, this world-first legislation to ban HFSS products in prominent locations is largely accepted. Most believed it will help consumers purchase and eat fewer unhealthy foods. This finding corresponds with other previous evidence demonstrating moderate-high levels of acceptance of obesity-related food policies in consumers and retailers [34–36], including one UK study of 7058 participants, where most found nudge and tax policy interventions on HFSS snack foods to be acceptable [37]. Research from Switzerland and Australia, however, suggests that more at-risk groups, such as individuals experiencing obesity or consumers of high-sugar drinks, are less supportive of food policies perceived to restrict personal liberties [38, 39]. Other reasons for not supporting food policies included the recognition that a single legislation ignores the complexity of food choice and that the availability and marketing of HFSS foods are ubiquitous. Commercial consumer polls in England suggest that 57% of consumers report they will continue to buy HFSS products after the legislation has been implemented, and only 40% believe it will achieve its aim [40]. These findings mirror comments from stakeholders in the current study and others [41] who have expressed concerns that the continued availability and marketing of HFSS foods, products and business exemptions and exploitation of legislation loopholes will weaken its health impact, particularly for those with the poorest dietary patterns.

### Policy implications

The results of our study included six policy recommendations. These consist of three for immediate action and three for medium-to-long-term action to map out a path for continual policy progress to tackle obesity through food system governance and targeted actions. Importantly, those for immediate action seek to address facets of the new legislation which stakeholders have identified as having scope for improvements. These include having reliable data on nutritional contents for food products, guaranteeing funds for enforcement and small business support. The three medium-to-long-term actions identify how complex system evaluation of the legislation enables the connection between food governance and health outcomes to progress towards the creation of a more sustainable and healthy food system.

The first recommendation for immediate action was for a central, government-authorised, HFSS calculator to be made freely available to all. It was voted the top priority amongst our stakeholders. This requirement is consistent with previous research which explored the views of six UK manufacturing and retail businesses about legislation implementation concerns related to the application of the 2004/2005 UK nutrient profiling model (NPM) [42]. Similar to issues raised in the current study, concerns included the complexity of identifying in-scope products, ensuring consistency in data between manufacturers and retailers, and discrepancies in businesses and enforcers having access to a NPM calculator. The High Court’s ruling in favour of the Department of Health and Social Care against Kellogg’s case that the NPM was flawed for its cereal products is indicative that this model will have the lasting application [43]. This ruling provides further rationale for the UK government to endorse and make freely available a single, central tool that will avoid needless duplication of resources and time to calculate product scores. It would also alleviate inconsistencies and confusion for businesses, enforcers and consumers [44].

Two further recommendations highlighted for immediate government action include the provision of extra ring-fenced resources for local authorities and targeted support for affected smaller businesses. Both of these recommendations are supported by previous evaluations of healthy eating initiatives in small food stores and independent takeaways [45–47]. Local authority staff responsible for enforcement of these food initiatives reported that they were most successful when appropriate financial and workforce resources were available. These resources ensured officers had dedicated time to establish strong, respectful relationships with businesses [45, 46]. Clear definitions and step-by-step guidance (as published by The Association of Convenience Stores [48]), accompanied by an intelligible rationale for local benefit (such as local childhood obesity and other health data), could be used in enforcement officers’ discussions with local businesses. Interactive stakeholder fora which bring businesses and other local stakeholders together to discuss practical issues around legislation could be more time efficient for local authorities whilst fostering a sense of community ownership [46, 47]. However, translation of resources into culturally appropriate languages is also likely to be necessary by local authorities to achieve consistent implementation, particularly amongst the convenience sector [45, 46].

Nonetheless, without additional investment from the national government, local authorities will have very limited capacity to enforce this legislation or support smaller businesses with implementation because budgets and staff are already stretched, and priority will rightly be given to threat-to-life enforcement activities (i.e. food allergies). The national government could consider providing incentives for small retailers and their suppliers to improve the healthfulness of the foods they offer, in recognition of their small profit margins and low customer demand for healthy foods [45, 49]. Previous scientific evidence suggests that government subsidies and engaging suppliers who can advise on and incentivise the stocking, handling and promoting of healthy items increases compliance with new food policies amongst small retailers [46, 50]. Given that the current UK cost-of-living crisis is likely to affect small businesses as much as the customers they serve, the need for financial support amongst small retailers in-scope of this legislation should be fully investigated to ensure they continue to be commercially competitive, particularly against their out-of-scope rivals which would help prevent health inequalities from widening further.

The two recommendations for medium-term government action—to conduct a robust evaluation and to refine the legislation to enhance public health objectives—are critical for legislation success and should be considered in tandem. Robust evaluation is of twofold importance: (i) evidence of effectiveness is likely to further increase legislation acceptance and compliance [36, 37, 50, 51] and (ii) identification of unintended consequences and why they occurred is critical to inform policy refinement to improve effectiveness rather than the policy being revoked. Independent evaluations that adopt a complex systems approach by academics and charities will ensure future refinements are in the best interests of the British population’s health [20]. As has been the case with tobacco policy [52], refinements to this policy play a role in prompting businesses to take further action to promote healthy retail environments. Of particular concern to stakeholder groups in the present study was the widening of dietary and health inequalities as a result of product and business exemptions, legislation loopholes and the affordability gap between healthy and unhealthy foods. Additionally, recent food retailer reports indicate that sales of alternative high-margin products such as beer, wine, spirits and vaping products will likely increase [53, 54]. Collectively, these findings highlight the need for future evaluations to adopt a complex systems perspective, incorporating both process and impact evaluation strategies, drawing upon existing complex systems frameworks and using mixed-methods research approaches [21, 55]. In the short-term, implementation factors related to changes to in-store sales of HFSS, alternative products and promotional strategies used and the impact on household grocery spend will be important to assess to provide insight into initial changes within the focal points of the system effected by the legislation. Mid- to long-term assessments of the effects on obesity levels, societal attitudes, business outcomes and broader alterations to the food system will enable an understanding of how collective changes produce emergent systems change. A robust evaluation should also cover qualifying and exempt businesses, particularly in the convenience sector (because of the higher preponderance and use of convenience stores in deprived areas [5, 8]) and assess differences by customer and regional demographics.

The final recommendation is for the development of a long-term strategic roadmap for food and health that sets out the suite of policies needed with anticipated timescales over the next 20–30 years to achieve the collective desire for a food system that ensures healthy, sustainable diets for all. Health and enforcer stakeholders participating in this study expressed the need for the government to more clearly articulate how this legislation fits within their broader strategy to address poor diet, obesity and health inequalities. Businesses spoke of the need for clarity on government’s food policy priorities so they can set their strategic and resource plans accordingly, whilst consumers wanted government to rebalance the price inequality between healthy and unhealthy foods where it is significantly more costly to fill up on healthier food than HFSS products. The recently published UK government food strategy [56] does not address these issues, despite the provision of concrete proposals for immediate action in Henry Dimbleby’s independent National Food Strategy review [1]. This review was the result of extensive stakeholder consultation and evidence synthesis and was largely welcomed by health advocates. It provides a valuable starting point to develop a long-term roadmap outlining key recommendations to curb the ubiquity of HFSS products in the food system and beginning to address healthy food affordability barriers for low-income families.

Despite widespread acknowledgment of the significant changes and governance needed for the UK food system to meet their own obesity and climate commitments, the UK government is yet to endorse the development of a long-term roadmap that can unify stakeholders’ priorities and timescales for the required action. Such a road map should build on work invested in Dimbleby’s plan and the Obesity Health Alliance’s 10-year healthy weight strategy [2], as well as consider additional strategies. Examples include incremental formulation targets for fibre, vegetables and fruit that are mandated for specified high-volume categories, phased reduction in marketing of ultra-processed foods or progressively increasing sector-specific targets for sales of minimally processed healthy foods. Aligned with Dimbleby’s final recommendation, such a long-term roadmap could be accompanied by the development of five yearly action plans with interim targets that are reviewed annually [1]; the statutory and independent Food Standards Agency positions themselves as being well-placed to fulfil such a role [57].

### Strengths and limitations of this study

To the authors’ knowledge, this study provides a first independent assessment combining four different stakeholder groups who form part of the system within which this legislation sits. Another strength is the rapid qualitative approach which allowed the inclusion of a range of views from 108 participants. The study also used a novel pre-implementation complex systems approach to provide policymakers with early insights into potential implementation pitfalls which could be addressed to facilitate effective implementation and enforcement and achievement of the intended impact. The sampling approach applied in this study could be considered a design weakness because the consumer sample did not include men, adolescents or older adults and the recruitment approach may have biased views from stakeholders with particularly strong opinions on this legislation. The study sample did, however, include participants who opposed this HFSS legislation, as well as those who held more favourable views and covered perspectives from different English regions and various sectors within the effected system. The timing of the interviews may have affected the responses given by participants. Consumers were interviewed before the second public consultation on the legislation had occurred and other stakeholders were interviewed before the detailed guidance was published. All findings presented in this paper, however, were validated with our customer PPI panel and with various business, enforcement and policymaker stakeholders in May 2022 which followed the publication of key related documents. Our study focused on the in-store aspects of the placement and promotion legislation but future research could explore perceptions, benefits and consequences of online implementation of the restrictions.

## Conclusions

Stakeholders affected by the upcoming placement legislation, including customers, businesses, enforcers and health advocates, are hopeful that it will improve customer purchases and diet. But, successful implementation and enforcement are contingent on three immediate-term government actions being met. These include (i) making a central HFSS calculator freely available to all, (ii) providing greater support for smaller businesses and (iii) providing ring-fenced resources to local authorities. Health advocates can conduct independent robust complex systems evaluations to assess both the implementation of the legislation (focusing particularly on assessing potential unintended outcomes) and the intended health outcomes to support the refinement of this important solid start to food system governance. Further development of a long-term roadmap that enables coherent policy initiatives across the food system with the unified goal of equitable, sustainable and meaningful dietary health is essential.

## Supplementary Information

**Additional file 1.** Interview questions for each stakeholder group.

**Additional file 2.** Sub-groups for each stakeholder group that participated in semi-structured interviews.

## Data Availability

Data described in this manuscript that have been collected by the research team during this study, and that can be anonymised, can be made available upon reasonable request to the corresponding author pending approval.

## Abbreviations

HFSS: High fat, sugar and salt
NPM: Nutrient profiling model
PPI: Patient and public involvement
RAP sheet: Rapid Assessment sheet
WRAPPED study: ‘Women’s Responses to Adjusted Product Placement and its Effects on Diet’ study
