# Supplementary material for: UK government’s new placement legislation is a ‘good first step’: a rapid qualitative analysis of consumer, business, enforcement and health stakeholder perspectives
Source: BMC Med. 2023 Jan 26;21:33. doi: 10.1186/s12916-023-02726-9 (PMC9878939; doi:10.1186/s12916-023-02726-9)
Supplement: Supplementary file 1 — Additional file 1. Interview questions for each stakeholder group. [file 12916_2023_2726_MOESM1_ESM.docx]

**Additional File 1**

Interview questions for each stakeholder group

| Stakeholder interview questions |
| --- |
| **Consumer interview schedule**   1. How tempted are you to buy products in prominent location? [i.e., i) at the store entrance, ii) on end of aisle displays, iii) at checkouts]  - *What type of food products do you usually buy in these locations*?  1. How do you think the location of products on the shelf affects your food shopping decisions?  - *What shelf location (bottom, mid, high) do you buy from most often?*  1. How do price promotions, such as multibuy promotions like two for one offer or 50% extra for the same price, affect your food shopping decisions?  - *What type of food products do you usually buy on price promotion?*   The British government is planning to bring in legislation to restrict some marketing strategies in supermarkets being used on unhealthy foods, in particular banning unhealthy foods from being located at the store entrance, end-of-aisles and checkouts and from being on price promotion.   1. What do you think about these restrictions being introduced (on high fat, sugar and salt products)? 2. How do you think these restrictions on unhealthy foods will affect your shopping habits? 3. What products would you like to see in prominent locations or on price promotions in supermarkets? 4. What responsibility do you think supermarkets and the government have to support customers to buy healthier foods?  - *What do you think is the role of individual, supermarkets and government in supporting customers make healthier food choices?* |
| **Business interview schedule**   1. What is your business’ current practice for promoting products in-store?  - *How has it changed in recent years?*  1. What is your opinion on the upcoming legislation?  - *How clear is the upcoming legislation?*  1. How do you foresee the legislation being enforced? 2. In what ways has your business started preparing for the upcoming legislation?  - *Changing in-store layout/online layout* - *Testing the impact legislation may have on your business* - *Challenges in complying with the legislation*  1. What changes will need to be made on your in-store/ online promotional strategies?  - *Changes in range of products on multibuy promotions* - *Changes in the type of product being promoted in prominent places* - *Altered promotion and placement of products deemed unhealthy by the legislation definitions*  1. What preparations have you made with your suppliers/manufacturers?  - *Impact on relationships with manufacturers of high fat, sugar and salt products*  1. What concerns do you have about the impact of the legislation on your business?  - *Impact on sales/ profits both online and in-store* - *Impact on existing or new customers*  1. Are there any areas where you feel your business could benefit from government support?  - *Support from local enforcement officers?*  1. What unintended impacts do you foresee could result from this legislation? 2. What do you think the benefits of the legislation will be?  - *Possible benefits* *for customers, businesses, society, government* |
| **Enforcer interview schedule**   1. What are your opinions of the legislation for restricting the promotion and placement of unhealthy foods (high fat, sugar and salt products) in retail store outlets?    - *Opinions on legislation for online retail*    - *Opinions about the need for the legislation*    - *Main benefits and/or concerns* 2. How clear is the upcoming legislation? 3. What preparations have your local authority made to enforce this legislation from October 2022?    - *Links made with other authorities to collectively approach enforcement of this legislation*    - *What tools/information will be used to assess compliance with the proposed definitions?*    - *Enforcement approach for smaller and larger outlets and for online outlets*    - *How will non-compliance be dealt with?*    - *How will fines be issued?* 4. What concerns do you have about enforcing this legislation? (i.e. in-store and online)    - *Possible ways to overcome the concerns* 5. How do you think businesses are responding to this intended legislation?    - *Impact on retailers?* 6. How well do you foresee retailers complying with this legislation in store?    - *Differences across regions and by outlet type and size* 7. How well do you foresee retailers complying with this legislation online? 8. What impact do you think the legislation may have on your local community?    - *Impact on consumers, society and government* 9. How will the enforcement of this legislation be accommodated within the existing workload of your team?    - *Capacity concerns for ongoing implementation of the legislation enforcement*    - *Additional resources to assist with enforcement (e.g. from professional bodies, central government)* 10. What, if any, support would be helpful for the enforcement of this legislation?     - *Financial support/equipment from central government*     - *Guidance/case studies* |
| **Health group interview schedule**   1. What are your opinions of the legislation for restricting the promotion and placement of unhealthy foods (high fat, sugar and salt products) in retail store outlets?    - *Opinions on legislation for online retail*    - *Main benefits and/or concerns* 2. How clear is the upcoming legislation? 3. How well do you think this legislation will be enforced?    - *How might this differ across local authorities/regions?*    - *Effectiveness of fines in preventing non-compliance*    - *Workload issues for enforcers thus limiting effectiveness of enforcement* 4. In your opinion, how could the enforcement of this legislation be supported? 5. What impact do you think the legislation will have on public health?    - *Unintended consequences for consumers, society or the government*    - *Public health impact changing overtime* 6. What impact do you think the legislation will have on businesses? 7. What suggestions do you have to effectively measure the impact of this legislation?    - *How might this differ across retailers, online/in-store etc?* 8. How do you think consumers will respond to this legislation?    - *What differences in consumer response do you predict across demographic groups?*    - *Different ethnic groups responses to the legislation; Gender; Socioeconomic groups* 9. What improvements do you think could be made to this legislation to maximise the public health benefit, particularly to vulnerable groups? |
