# Supplementary material for: UK government’s new placement legislation is a ‘good first step’: a rapid qualitative analysis of consumer, business, enforcement and health stakeholder perspectives
Source: BMC Med. 2023 Jan 26;21:33. doi: 10.1186/s12916-023-02726-9 (PMC9878939; doi:10.1186/s12916-023-02726-9)
Supplement: Supplementary file 2 — Additional file 2. Sub-groups for each stakeholder group that participated in semi-structured interviews. [file 12916_2023_2726_MOESM2_ESM.docx]

Additional File 2

Tables showing sub-groups for each stakeholder group that participated in semi-structured interviews

Additional File 2: Table S1 – Consumer Characteristics

| Consumers (n=34) | |
| --- | --- |
| Women, n (%) | 34 (100%) |
| Age (years), median (IQR) | 35.7 (31.7, 39.4) |
| White Ethnicity, n (%) | 27 (79%) |
| Married, n (%) | 19 (61%) |
| Low education (no qualifications beyond age 16), n (%) | 17 (50%) |
| Most deprived half of area deprivation (IMD), n (%) | 10 (29%) |
| Paid employment, n (%) | 19 (57%) |
| Pounds (£) spent on food per week, median (IQR) | 70 (45, 100) |

Additional File 2: Table S2 – Business Group Characteristics

| Businesses (n=24) | Role type, number (%) | | | | |
| --- | --- | --- | --- | --- | --- |
| *Retailers* | **Trade body**  **(3 bodies, n=3)** | **Supermarkets**  **(3 stores, n=4)** | **Convenience Stores**  **(3 stores, n=3)** | **Online Retailer**  **(1 chain, n=2)** | **Non-food retailers**  **(2 chains, n=3)** |
| Director/Chief Exec | 3 (100%) | 0 (0%) | 0 (0%) | 0 (0%) | 0 (0%) |
| Senior Manager (e.g. head of) | 0 (0%) | 2 (50%) | 1 (33.33%) | 2 (100%) | 2 (66.66%) |
| Manager (e.g. health/store manager) | 0 (0%) | 1 (25%) | 1 (33.33%) | 0 (0%) | 1 (33.33%) |
| Professional/other (e.g. nutritionist, co-ordinator) | 0 (0%) | 1 (25%) | 1 (33.33%) | 0 (0%) | 0 (0%) |
| *Manufacturers and wholesalers* | **Trade body**  **(2 bodies, n=2)** | **Manufacturers**  **(5 companies, n=6)** | **Wholesalers**  **(1 wholesaler, n=1)** |  |  |
| Director/Chief Exec | 1 (50%) | 0 (0%) | 0 (0%) |  |  |
| Senior Manager (e.g. head of) | 0 (0%) | 3 (50%) | 0 (0%) |  |  |
| Manager (e.g. health/store manager) | 1 (50%) | 3 (50%) | 0 (0%) |  |  |
| Professional/other (e.g. nutritionist, co-ordinator) | 0 (0%) | 0 (0%) | 1 (100%) |  |  |

Additional File 2: Table S3 – Enforcement Officer Characteristics

| Enforcement Officers (n=22) | Trading Standards officers (n=13) | Environmental health officers, (n =6) | Public Health officers, (n =3) |
| --- | --- | --- | --- |
| *Job Role* number (%) |  |  |  |
| National representative/Food lead | 1 (8%) | 1 (17%) | 0 (0%) |
| Senior Officer | 7 (54%) | 2 (33%) | 1 (33%) |
| Officer | 5 (38%) | 3 (50%) | 2 (67%) |
| *Total* | *13 (100%)* | *6 (100%)* | *3 (100%)* |
| *Trading standards providing primary authority support to retailers? % (n)* | | | |
| Yes | 5 (38%) | N/A | N/A |
| No | 8 (62%) | N/A | N/A |
| *Total* | *13 (100%)* | N/A | N/A |
| *Region* |  |  |  |
| North | 3 (23%) | 2 (34%) | 0 (0%) |
| Central | 2 (15%) | 1 (16.25%) | 0 (0%) |
| South | 8 (62%) | 1 (16.25%) | 2 (67%) |
| London Borough | 0 (0%) | 1 (16.25%) | 1 (33%) |
| Wales | 0 (0%) | 1 (16.25%) | 0 (0%) |
| *Total* | *13 (100%)* | *6 (100%)* | *3 (100%)* |

Additional File 2: Table S4 – Health Representatives Characteristics

| Health Representatives (n=28) | Role type, number (%) | |
| --- | --- | --- |
| *Public Health/Nutrition Academics (n=9)* | | |
| Professor/Associate Professor | | 4 (44%) |
| Research Fellow/Associate | | 4 (44%) |
| Lecturer | | 1 (12%) |
| *Non-Government Charities and Royal Societies (n=19)* | | |
| Director/CEO | | 6 (32%) |
| Senior manager (e.g. head of campaigns) | | 2 (10%) |
| Manager (e.g. of policy, health) | | 5 (25%) |
| Professional/other (e.g. nutritionist, campaign coordinator) | | 6 (32%) |
